# The prevalence of misophonia in a representative population-based survey in Germany

**DOI:** 10.1007/s00127-024-02707-0

**Published:** 2024-07-04

**Authors:** Elisa Pfeiffer, Marc Allroggen, Cedric Sachser

**Affiliations:** Clinic for Child and Adolescent Psychiatry/Psychotherapy, Ulm University, Steinhoevelstr. 5, 89075 Ulm, Germany

**Keywords:** Misophonia, Prevalence rate, Adult, General population

## Abstract

**Purpose:**

Misophonia is a new disorder, currently defined as significant emotional and physiological distress when exposed to certain sounds. Although there is a growing body of literature on the characteristics of the disorder, the prevalence in the general population is still relatively unknown. This study therefore aims at determining the prevalence and symptom severity of misophonia in a large and representative general population sample in Germany.

**Methods:**

To examine the prevalence of misophonic sounds, misophonic reactions and misophonia severity, a cross-sectional population representative survey in Germany has been conducted. Participants (*N* = 2.522) were questioned retrospectively about misophonic symptoms using the Amsterdam Misophonie Scale – Revised (AMISOS-R).

**Results:**

Overall 33.3% reported to be sensitive to at least one specific misophonic sound. Within the total sample, subthreshold symptoms were reported by 21.3%, mild symptoms were reported by 9.9%, moderate to severe symptoms were reported by 2.1%, and severe to extreme symptoms were reported in 0.1% of participants.

**Conclusion:**

Based on the diverging presentations and prevalence rates of misophonic sounds, reactions and symptoms according to the severity, it seems worthwhile to conceptualize misophonia as a rather continuous spectrum disorder (subthreshold, mild, moderate to severe), still taking into account that an additional categorical diagnostic approach might be necessary to derive a diagnosis in clinical practice.

## Background

The phenomenon misophonia was first mentioned in 2001 by Pawel and Margaret Jastreboff in the context of hyperacusis and tinnitus research [1, 2]. Misophonia is typically described as a distinct clinical disorder of decreased tolerance for specific auditory stimuli resulting in specific physiological and emotional reactions (e.g. anger, disgust) upon exposure to such sounds. These misophonic sounds (often referred to as “misophonic triggers”) mostly include sounds which are directly or indirectly made by other people (e.g. chewing or scratching). These repeatedly occurring sounds are usually not excessively loud [3] and are not limited to the family environment, but can be especially strong by a person one knows well (e.g. a spouse) [4]. Misophonia can result in significant impairment of daily functioning [5] as well as a decrease in quality of life [6].

Despite growing interest, misophonia remains relatively understudied. The disorder has not yet been classified as a mental, neurological or auditory disorder. There is, however, a strong and growing interest in misophonia among psychologists and psychiatrists, auditory neuroscientists, audiologists, and occupational therapists. Especially due to the high rates of psychiatric comorbidity [7–10], several researchers have made a case for misophonia to be classified as a mental disorder [11]. While scholars recognize misophonia’s clinical features and underlying neurophysiological mechanism [5], its definition varies due to diverse criteria and assessment methods. Two prominent sets of criteria, proposed by different groups, include observations and clinical interviews with large samples [4, 12], and interviews with self-identified misophonia sufferers [13]. Despite similarities, both sets have faced criticism based on clinical observations [14]. In order to bring more cohesion to the growing field of researchers and clinicians who are interested in getting a better understanding of misophonia, a recent Delphi study [15], which conducted a systematic literature review and engaged in a consensus-building process, involving an expert committee (15 members), ought to synthesize the current findings and compile a definition of the disorder which includes both previous conceptualizations. Their consensus definition includes a general description of the disorder, as well as a description of the specific triggers and reactions to misophonic triggers, the influences on misophonic reactions and the functional impairment due to the disorder. However, this new definition and the Delphi process itself have already been criticized by another scholar in the field [16]. The criticism of Brout is mainly related to the focus of the consensus definition on observable behavior and recommends a definition with a more dimensional approach. In sum, misophonia is a relatively new disorder, the conceptualization of which was well elaborated in the Delphi study, but may still focus mainly on observable behavior. Therefore, prevalence studies play a crucial role in providing insights into the frequency or occurrence of the phenomenon within a specific population, thereby contributing to a deeper understanding of the disease burden.

The prevalence rates of misophonia have mostly been described in college student [17] and clinical samples [18] or with online surveys in the general population [19]. Throughout these studies, misophonic triggers and symptoms were assessed inconsistently with different assessment tools, with heterogeneous underlying concepts of the disorder, resulting in very differing prevalence rates [10]. For example, a study in China reported misophonic symptoms in 6% of their college student sample [20], whereas another study in the US reported clinically significant misophonia symptoms in nearly 20% of the students [21], and in a study with undergraduate students in the UK even 49.1% of the participants in their online survey reported misophonic symptoms (AMISO-S questionnaire; [17]).

The research on the prevalence of misophonia triggers and symptoms in the general population is expanding, but still there only a few studies. In the following we will focus on three population-based studies that investigated the prevalence of misophonia which were conducted in Ankara, Turkey [14], in the UK [22] and in Germany [23]. The authors in the study in Turkey developed a misophonia interview and assessed *N* = 541 residents as a representative and randomized community sample. The proposed diagnostic criteria diagnosed 12.8% of their participants with misophonia. They found that several variables such as female gender, being single and being younger were significantly related to misophonia, but only age, a family history of misophonia and past contact with mental health services predicted misophonia. In the study conducted in UK, participants of the third wave (*N* = 800) were recruited via the platform “prolific.com” and constituted a representative sample of the UK general population. The authors used the “Selective sound sensitivity syndrome scale” (S-Five; [24] and reported a prevalence of 18% of bothersome misophonia in their sample. Lastly, the German study [23], which is also the most recent study (data was collected between December 2020 and March 2021), found a prevalence of participants with clinically relevant scores in the AMISOS-R of 5.9% (caution: the clinical cut-off score was self-developed, there is currently no validated score). Additionally, 2.2% of individuals fulfilled the cut-off points in both their measures. In sum, these population-based studies present a range of prevalence rates, likely attributed to variations in measurement methods, recruitment approaches, and sampling techniques, which may limit their comparability. This emphasizes the importance of replication studies and the need to develop a nuanced understanding of symptom severity. Instead of relying solely on the thresholds, it is essential to explore the varying degrees of symptom severity within the general population and replicate studies using consistent measurement tools.

### Study aim

The aim of this study was to determine the prevalence and degrees of severity of misophonia in a large and representative general population sample in Germany, using the most frequently used assessment tool AMISOS-R to allow for comparisons with other population-based studies, as well as with student and convenience samples. Additionally, this study aims at determining prevalence differences of the misophonic symptoms with regard to sociodemographic (e.g. gender, age) and psychosocial (e.g. migration background) variables, and potential differences in symptoms of depression and anxiety regarding misophonia severity in an exploratory manner. Finally, the study will explore potential psychosocial and demographic factors, which might influence misophonia severity in an exploratory manner. Hence, with the results of this study, we would like to contribute to our current understanding of misophonia and stimulate more epidemiological research on the distribution of the disorder in the general population.

## Methods

### Procedure and sample

In cooperation with a professional demographics research institute (USUMA) a representative sample of the German population (age 16–96) was collected employing a random route approach. In a first step, 258 German regional areas were predefined using the reference system for representative studies in Germany provided by the “ADM-Sampling- System”. Next, the target households within these regional areas were selected according to a random route procedure. For multi-person households, one person was randomly selected using the Kish selection grid technique. Following these steps, possible participants were not recruited from specific platforms, but were unexpectedly contacted directly by trained assessors of the research institute USUMA. Demographic data was assessed face-to-face by a trained interviewer and the participants alone filled out other instruments like the AMISOS-R or PHQ-4, while the interviewer was waiting in the room. The population-based survey was conducted in the period from 3th March to 26th May 2022. The authors assert that all procedures contributing to this work comply with the ethical standards of the relevant national and institutional committees on human experimentation and with the Helsinki Declaration of 1975, as revised in 2008. All procedures involving human subjects/patients were approved by the Ethics Committee of the University of Leipzig (594/21-ek). Written and verbal informed consent was obtained from all respondents, who indicated their willingness to take part in the study. Anonymity in responses was guaranteed by deleting the link between the study code and the name of the participant after data entry. To qualify for inclusion in the survey, participants had to be at least 16 years of age and have sufficient German language skills. Prospective subjects were informed that the study was about psychological health and well-being. Out of the 6192 addresses used, 2522 responded indicating a utilization rate of 41.2%. The final sample consisted of *N* = 2522 respondents (51.4% female) with an average age of *M* = 49.42 years (*SD* = 18.89; range 16–96). A detailed description of the sample with means and standard deviations regarding different sociodemographic factors and misophonia severity can be found in Table 1.

**Table 1:** Sociodemographic characteristics of the total sample and the comparison of the misophonia subsample severity categories according to the AMISOS-R

	Total Sample (*N* = 2.522)	Misophonia Subsample (*n* = 844)			Statistic
		Subthreshold Misophonia (*n* = 537)	Mild Misophonia (*n* = 251)	Moderate to extreme Misophonia (*n* = 56)	
Age	49.42	50.53 (18.63)	47.57 (18.68)	48.71 (17.95)	*X*^2^ (2) = 3.43, *p* = 0.180
Gender					
*Female*	51.4	59.8	62.0	53.6	*X*^2^ (2) = 1.37, *p* = 0.503
*Male*	48.4	39.9	38.0	46.4	
*Divers*	0.2	0.4	0	0	
Migration background	13.2	13.2	13.9	14.3	*X*^2^ (2) = 0.11, *p* = 0.948
German citizenship	96.3	97.2	98.4	94.6	*X*^2^ (2) = 2.76, *p* = 0.252
Living with partner	53.4	55.3	57.1	50.9	*X*^2^ (2) = 0.70, *p* = 0.703
Household net-income	2.056,17 (983,86)	2.046,83 (955,46)	1.920,15 (830,42)	1.851,73 (1.026,12)	*X*^2^ (2) = 3.86, *p* = 0.145
Religion					*X*^2^ (4) = 0.60, *p* = 0.964
*Protestant*	35.8	35.9	36.0	32.7	
*Catholic*	30.6	28.9	27.2	30.9	
*Muslim*	2.8	1.9	0.8	0.0	
*Other*	1.9	1.1	4.4	0.0	
*No confession*	28.9	32.1	31.6	36.4	
PHQ-4 Total Score	1.46 (2.15)	1.54 (1.91)	2.80 (2.64)	4.29 (3.55)	*X*^2^ (2) = 69.88, *p* < 0.001
PHQ-2 Depression Score	0.80 (1.19)	0.82 (1.10)	1.44 (1.46)	2.25 (1.91)	*X*^2^ (2) = 61.87, *p* < 0.001
GAD-2 Anxiety Score	0.66 (1.10)	0.72 (1.00)	1.35 (1.38)	2.04 (1.76)	*X*^2^ (2) = 65.76, *p* < 0.001

*Note* Values are means (standard deviations) or %, as appropriate

As the residuals of age, household net-income, PHQ-4, PHQ2 and GAD-7 scales were not normally distributed, Kruskal-Wallis-Tests were used for comparisons

Based on the cell frequency of diverse gender smaller than 5, the *X*^2^-test was only used comparing male vs. female

Based on the cell frequencies of Muslim religion and category other smaller than 5 the *X*^2^-test was only used comparing protestant vs. catholic vs. no confession

#### Instruments

##### Sociodemographic information

Sociodemographic information was collected within an interview format assessing gender, age, migration background (question on whether participants were born in Germany (yes/ no)), living with a partner and household net-income.

##### AMISOS-R

Misophonia symptoms were measured using the Amsterdam Misophonia Scale-Revised (AMISOS‐R: [4]). The first section of the questionnaire assesses specific misophonic triggers (e.g. eating sounds, nasal sounds, throat sounds, etc.) and the emotions which might be evoked when hearing the sounds (irritation, anger, disgust, or other) via two checklists. This part of the questionnaire is an addition to the former AMISO-S [12]. The second section of the AMISOS-R consists of 10 items (assessment period: 3 last 3 days) using a 4-point Likert scale with the anchors: 0 = “not”, 1 = “mild”, 2 = “moderate”, 3 = “severe”, 4 = “extreme” for 8 items. The range of the total scale of the AMISOS-R is 0–40, where higher scores indicate more severe symptoms: 0–10 = “normal to subclinical misophonia”; 11–20 = “mild misophonia”; 21–30 = “moderate to severe misophonia”; 31–40 = “severe to extreme misophonia”. The items in the AMISOS-R have been modified compared to the AMISO-S (total of 6 items) and possible scores in the AMISO-S range from 0 to 24 (with higher scores also indicating more severe misophonia). The reliability of the AMISOS-R in the current sample was good (α = 0.89, interpreted according to Cronbach [25]).

##### PHQ-4

The Patient Health Questionnaire-4 (PHQ-4), consisting of the subscales PHQ-2 and GAD-2, is a widely used screening instrument for depressive and anxiety symptoms in the last two weeks, and is implemented in clinical settings and in epidemiological studies [26]. THE PHQ-4 consists of 4 items using a 4-point Likert scale with the anchors: 0 = “not at all”, 1 = “several days”, 2 = “more than half the days”, 3 = “nearly every day”. The range of the total scale of the PHQ-4 is 0–12, where higher scores indicate more anxiety and depression symptoms (95%-thresholds at a score of 6–7). The range of the GAD-2 and PHQ-2 is 0–6, with 95%-thresholds at a score of 3–4 respectively [26]. The reliability (interpreted according to Cronbach [25]) of the total scale in the current sample was good for the PHQ-4 (α = 0.88), the GAD-2 (α = 0.83) and the PHQ-2 (α = 0.79).

#### Statistical analyses

First, descriptive statistics of the demographic variables, prevalence rates of misophonic sounds, misophonic reactions and misophonia severity were calculated regarding the full sample and the subsample, which reported sensitivity towards sounds. Second, within the misophonia severity categorization of the AMISOS-R, demographic characteristics (age, gender, migration background, living with partner, household net-income, religion) as well as symptoms of depression and anxiety were inspected and compared. χ^2^-tests were used for categorical variables with cell counts higher five. For the dimensional variables (age, household net-income, depression and anxiety) Kruskal-Wallis-tests were used for comparison, as residuals were not normally distributed. Third, multiple regression analysis was used to examine possible demographic and psychosocial predictors (gender, age, migration background, living with partner and household net-income, religion) of misophonia symptom severity. Multicollinearity was not a problem for our independent variables (Tolerance values for predictors: 0.93–0.99; VIF values for predictors: 1.01–1.08). The expected mean error of the regression model was zero and the residuals were not systematically correlated with explaining variables. The variance of errors was constant. Although Kolmogorov-Smirnov-Test was significant, which is the case also by small deviations in large samples, visual inspection showed an approximate normal distribution of residuals. The Breusch-Pagan test (χ^2^ (1) = 56.37; *p* < 0.001) and visual inspection of scatter plot (x-axis: regression standardized predicted value; y-axis: regression standardized predicted value) revealed heteroscedasticity. To address the problem of heteroscedasticity, parameter estimation with robust heteroskedasticity-consistent standard errors was performed. To additionally address high leverage points (outliers) the HC4 estimator was used [27]. All analyses were performed using IBM^®^ SPSS^®^ Statistics version 29.

## Results

### Misophonic sounds

Overall 66.2% (*n* = 1670) reported to have no particular sensitivity towards misophonic sounds according to the AMISOS-R in comparison to other people. Among the categories of misophonic sounds, 21.7% (*n* = 547) of the participants reported to be more sensitive to eating sounds, 13.4% (*n* = 338) to repeating clicking/ tapping sounds, 9.6% (*n* = 243) to nasal sounds, 7.0% (*n* = 175) to ambient sounds; 5.6% (*n* = 142) rustling sounds; 5.4% (*n* = 135) to mouth/ throat sounds, and 2.2% (*n* = 54) to specific sounds.

### Misophonic reactions

Among those participants who reported at least one specific misophonic sound (33.5%, *n* = 844), 74.4% (*n* = 627) reported irritation, 20.3% (*n* = 172) reported anger, 29.0% (*n* = 245) reported disgust as emotional misophonic reactions to the misophonic sounds. Other misophonic reactions were reported by 4.1% (*n* = 35) and included nervousness, agitation, anxiety, goosebumps, sleeping problems and concentration problems.

Among those participants who reported sensitivity to misophonic sounds, 60.2% (*n* = 495) reported to not think or be exposed to the misophonic sounds, 31.7% (*n* = 267) reported to think or be exposed less than one hour per day to the misophonic sounds, 6.2% (*n* = 52) reported to think or be exposed one to three hours per day to the misophonic sounds, 0.7% (*n* = 6) reported to think or be exposed three to six hours per day to the misophonic sounds, 0.1% (*n* = 1) reported to think or be exposed more than eight hours per day to the misophonic sounds. 2.6% (*n* = 22) of the participants who reported to be sensitive to particular sounds have not answered the question on time being exposed or thinking about the misophonic sounds.

### Misophonia symptoms and severity

In the total sample of *N* = 2522 participants, subthreshold symptoms were reported by 21.3% (*n* = 537), mild symptoms were reported by 9.9% (*n* = 251), moderate to severe symptoms were reported by 2.1% (*n* = 53) and severe to extreme symptoms were reported in 0.1% (*n* = 3) of participants. Among those participants who reported misophonic sounds (*n* = 844), this reflected 63.6% with subthreshold symptoms, 29.7% with mild symptoms, 6.2% with moderate to severe symptoms and 0.4% with severe to extreme symptoms. The AMISOS-R mean score in those participants reporting misophonic sounds (*n* = 844) was *M* = 9.35 (*SD* = 6.5; range = 0–34).

### Predictors of misophonic symptom severity

A multiple linear regression model was calculated to predict the misophonia symptom severity (AMISOS-R total score) in the subsample reporting misophonic sounds based on variables gender, age, migration background, living with partner and household net-income. A significant regression equation was found (*F*(5,835) = 2.98, *p* = 0.011), with a poor prediction (goodness of fit) of *R*^2^ of 0.018. Only age (β = − 0.028, *p* = 0.013) and household net-income (β = − 0.0.001, *p* = 0.004) were significant predictors of misophonia symptom severity in the way that higher age was associated with lower misophonia symptoms and higher household net income was associated with lower misophonia symptoms (Table 2).

**Table 2:** Multiple linear regression model with misophonia symptom severity as dependent variable and gender, age, migration background, living with partner and household net-income as independent variables

Predictor	b	SE	CI 95% lower	CI 95% upper	t	*p*
(Constant)	11.743	0.868	10.039	13.447	13.527	0.000
Gender	0.104	0.448	-0.775	0.983	0.232	0.817
Age	-0.028	0.011	-0,050	-0.006	-2.483	0.013
Migration background	0.565	0.681	-0,772	1.901	0.829	0.407
Living with partner	0.177	0.456	-0,718	1.072	0.388	0.698
Household net income	-0.001	0.000	-0,001	0.000	-2.860	0.004

*Note R*^2^ = 0.018; SE = Standard Error; CI 95% lower and upper represent the lower-limit and upper-limit of the unstandardized b regression weights

## Discussion

The aim of this study was to describe misophonia in a large representative population-based sample in Germany to provide the field with new insights on the distribution and phenomenology of misophonia in the general population.

Altogether 34% of the participants reported to be sensitive to any misophonic sound. In line with a study by Jager et al. (2020) [4], with subjects with misophonia symptoms who were referred to mental health services, the most frequent misophonic sounds were eating, nasal and repetitive clicking/ tapping sounds. The dominance of these sound categories has also been seen in online surveys with non-clinical samples [13, 19, 28]. The most frequently reported misophonic emotional reaction, among those participants who reported at least one specific misophonic sound, was irritation and to a lesser extent anger and disgust, which are also specifically named in the misophonia criteria by Jager et al. (2020) [4] and the Delphi definition [15].

The mean score in those participants reporting misophonic sounds was three times lower compared to the Dutch clinical sample (*M* = 29.78) which also completed the AMISOS-R [4]. Mean scores in the AMISOS-R in the previous German population-based study, which has used almost the same data collection strategy and a very similar population, were also higher compared with our sample. However, the study by Jakubovski and colleagues [23] did not report the specific mean score of participants who report misophonic sounds, which makes comparability more difficult.

In the general population, subthreshold misophonia was reported by 21.3%, to a much lesser extend mild (9.9%) or moderate to extreme symptoms (2.3%). This result is similar to the reported prevalence rate of clinically relevant symptoms in the previous German study (5-5.8%) which found significantly lower misophonia rates compared with earlier studies conducted in Turkey and UK. Hence, both German studies showed that prevalence rates of misophonia in the general population are not as high as previously reported (considering that this effect could potentially also be explained by methodological factors such as the usage of different measures or recruitment strategies). This finding indicates that being sensitive to specific sounds might be common, or even a personality trait, among healthy adults and does not necessarily indicate that a person suffers from clinically relevant misophonia. Instead there seems to be a large group of people who are sensitive to sounds and report some symptoms, but won’t fulfill misophonia criteria and might thus not need specific treatment. In line with this argument, we found in our data that only a very small rate of participants actually report clinically relevant symptoms (2.3%), but about every tenth person in our study reports any misophonic symptoms. Furthermore, in our subsample with misophonic sounds, prevalence rates on different severity degrees are much higher with 37.3% reporting at least mild symptoms of misophonia.

When looking at the results more closely, we found that only 39.8% of participants who reported misophonic triggers spend any time thinking or avoiding these sounds, and 2.6% of the participants who reported to be sensitive to particular sounds have not even answered the question on time being exposed or thinking about the misophonic sounds. Hence, many people might find the stimuli disturbing but might not bother enough to think about them more actively or to try to avoid them as a consequence. This finding is of particular interest for two reasons: Firstly, thinking about misophonic sounds is not included as a symptom of misophonia in any known criteria, so it might not sufficiently distinguish between people with and without misophonia. Secondly, people who suffer from misophonia normally cannot influence the frequency of exposure to these triggers as they are performed by others and depend on different circumstances/ context (e.g. chewing is normally more frequent during lunch hours). Hence, the general frequency of exposure to these triggers might not be related to the severity of misophonia symptoms, but instead only the individual’s perception of and reaction to the triggers. A recent study supports this by showing that the severity of misophonia symptoms is associated with worse cognitive control when exposed to misophonia trigger sounds [29]. Lastly, this lack of control of exposure to triggers might result in a certain learnt passivity of the individual and in people with strong misophonia symptoms to dysfunctional coping strategies (e.g. avoid eating with others).

Taken together, the results of this study highlight the need to look at different symptom severity groups more closely, instead of simple cut-off scores, as this might explain the differences in prevalence rates in previous population-based studies. Future research should investigate the different groups more closely regarding potential comorbid psychiatric symptoms or general sociodemographic characteristics to better understand the misophonia symptom profiles and their treatment altogether. Ultimately, our results support the idea towards are more dimensional, instead of a categorical, understanding of the disorder, similar to other psychiatric disorders such as autism spectrum disorder. However, abandoning categorical diagnoses in clinical practice and research altogether will not be beneficial for individuals suffering from severe misophonia, as a categorical diagnosis is required for treatment reimbursement and obtaining adjustments in work or school conditions in most countries Regarding psychiatric comorbidity, we found that participants reporting misophonia symptoms report higher levels of depression and anxiety, which is in line with previous studies on psychiatric comorbidities of misophonia [21, 30], indicating higher vulnerability for psychiatric symptoms in patients with misophonia.

The multiple linear regression model was significant but the sociodemographic and psychosocial factors explained only a very small amount of variance of the misophonia symptom severity in our sample, which is similar to the previous German based study (R^2^ = 0.18; [23]). The only significant predictors were age and household net-income, but also with very small effects. Therefore, we conclude that misophonia symptoms show no substantial association with the tested sociodemographic and psychosocial factors and can therefore be seen as a universal phenomenon that permeates all levels of society. This finding also raises the question on whether misophonia might instead have a potential genetic or even epigenetic pathomechanism. Recent studies [31, 32] also found preliminary evidence for the genetic aetiology of misophonia. For example, a study by Smit and colleagues found via clustering algorithms in an unpublished genome-wide association study that misophonia clustered strongly with other psychiatric disorders such as PTSD or anxiety (no correlation with ADHS, OCD, psychotic disorders) and specific personality profiles (neuroticism/ guilt and irritability/ sensitivity) [31]. Future studies should replicate and further investigate the potential genetic underpinnings of misophonia and consider these for future treatment plans.

## Limitations and future research

Despite several strengths of this research, such as the large sample size (larger compared with Jakubovski and colleagues [23]), several limitations need to be considered. Firstly, although the questionnaire assesses the most prominently described misophonic triggers, more elaborate trigger lists [4] might be used in future research to better understand the frequency and combinations of specific sounds, and to help discriminate those sounds with misophonic reactions from those without misophonic reactions. Secondly, due to the study design and assessment, only participants age 16 and older were included in this study, which is also the case in the study by Jakubovski et al. [23]. Especially since the onset of misophonia is most often in childhood/ adolescence [12, 19, 28], and there are only limited studies with children and adolescents on their misophonia symptoms [6], future research needs to include children and adolescents in population based surveys to better describe the phenomenology and clinical correlates of misophonia in children and adolescents. This might be especially important, as there is already some evidence that children with misophonia report poorer well-being and life satisfaction, compared with children without misophonia [6]. Thirdly, since the AMISOS-R is commonly utilized in misophonia research, we opted to employ this measure to enhance comparability with other studies. However, it’s important to note that this measure (in both Dutch and German versions) has not undergone sufficient validation. Despite its lack of validation, it has shown a strong correlation with validated measures, for example the correlation between MQ and AMISOS-R *r* = 0.72, *p* < 0.01 [23]). Future studies might assess misophonia in light of the newly proposed criteria in the Delphi process. Future steps after the Delphi process will be to discuss these new criteria within the scientific community and to then develop and validate a measure based on the final criteria. Fourthly, although the PHQ-4 is a widely used and validated measure for depression and anxiety (GAD-7 items), it might not capture the entire conceptualization of the disorders. Additionally, it would have been interesting to further investigate the overlap of misophonia with other internalizing disorders such as posttraumatic stress disorder (PTSD) or obsessive compulsive disorder, as both disorders show similarities with misophonia [10]. Lastly, since misophonia is a rather new discovered phenomenon, it might be helpful for the field to gather more qualitative data on the patient’s experiences and learn from their individual perspective on the symptomatology. Within quantitative research, (semi-structured) interviews might provide the field with a more thorough description of the symptoms, compared with standardized questionnaires [14].

## Conclusions

In the absence of a common understanding of the disorder, resulting in different diagnostic criteria and assessment tools, this epidemiological study might shed more light on misophonic sounds and symptoms in the general population and stimulate more population-based surveys in other countries to better understand different cultural and societal influences on the disorder. Based on the diverging prevalence rates of misophonia triggers and symptom severity scores in this study, and the comparably low percentage of people spending time to avoid misophonic triggers at all, it seems worthwhile to consider misophonia as a somewhat dimensional disorder after all.

## Data Availability

The data is available upon request from the authors.

## Abbreviations

AMISO-S: Amsterdam misophonia scale
AMISOS-R: Amsterdam misophonia scale revised
UK: United Kingdom
USUMA: Unabhängiger service für umfragen, methoden und analysen (German company name)
PHQ-4: Patient health questionnaire-4
GAD-2: Generalized anxiety disorder 2-item

## References

[1] Jastreboff MM, Jastreboff PJ (2001) Components of decreased sound tolerance: hyperacusis, misophonia, phonophobia. ITHS News Lett 2:1–5

[2] Jastreboff MM, Jastreboff PJ (2002) Decreased sound tolerance and tinnitus retraining therapy (TRT). Australian New Z J Audiol 24:74–84

[3] Jastreboff P, Jastreboff M (2014) Treatments for decreased sound tolerance (hyperacusis and misophonia). Semin Hear 35:105–120. 10.1055/s-0034-1372527

[4] Jager I, de Koning P, Bost T, Denys D, Vulink N (2020) Misophonia: Phenomenology, comorbidity and demographics in a large sample. PLoS ONE 15:e0231390 32294104 10.1371/journal.pone.0231390 PMC7159231

[5] Brout JJ, Edelstein M, Erfanian M, Mannino M, Miller LJ, Rouw R et al (2018) Investigating misophonia: a review of the empirical literature, clinical implications, and a research agenda. Front Neurosci ;36 10.3389/fnins.2018.00036 PMC5808324 29467604

[6] Rinaldi LJ, Smees R, Ward J, Simner J (2022) Poorer Well-being in children with Misophonia: evidence from the Sussex Misophonia Scale for adolescents. Front Psychol ;13 10.3389/fpsyg.2022.808379 PMC9019493 35465571

[7] Erfanian M, Kartsonaki C, Keshavarz A (2019) Misophonia and comorbid psychiatric symptoms: a preliminary study of clinical findings. Nord J Psychiatry 73:219–228 31066600 10.1080/08039488.2019.1609086

[8] Rosenthal MZ, McMahon K, Greenleaf AS, Cassiello-Robbins C, Guetta R, Trumbull J, Anand D, Frazer-Abel EF, Kelley L (2022) Phenotyping misophonia: Psychiatric disorders and medical health correlates. Front Psychol 13:1–22. 10.3389/fpsyg.2022.941898 10.3389/fpsyg.2022.941898 PMC9583952 36275232

[9] Siepsiak M, Rosenthal MZ, Raj-Koziak D, Dragan W Psychiatric and audiologic features of misophonia: use of a clinical control group with auditory over-responsivity. J Psychosom Res 2022;156. 10.1016/j.jpsychores.2022.110777 10.1016/j.jpsychores.2022.110777 35259551

[10] Ferrer-Torres A, Giménez-Llort L, Misophonia (2022) A systematic review of current and future trends in this emerging clinical field. Int J Environ Res Public Health 19:6790 35682372 10.3390/ijerph19116790 PMC9180704

[11] Taylor S, Misophonia (2017) A new mental disorder? Med Hypotheses 103:109–117. 10.1016/j.mehy.2017.05.003 28571795 10.1016/j.mehy.2017.05.003

[12] Schröder A, Vulink N, Denys D (2013) Misophonia: diagnostic criteria for a new psychiatric disorder. PLoS ONE 8:e54706 23372758 10.1371/journal.pone.0054706 PMC3553052

[13] Dozier TH, Morrison KL (2017) Phenomenology of misophonia: initial physical and emotional responses. Am J Psychol 130:431–438

[14] Kılıç C, Öz G, Avanoğlu KB, Aksoy S (2021) The prevalence and characteristics of misophonia in Ankara, Turkey: population-based study. BJPsych Open 7:1–6. 10.1192/bjo.2021.978 10.1192/bjo.2021.978 PMC8358974 34353403

[15] Swedo SE, Baguley DM, Denys D, Dixon LJ, Erfanian M, Fioretti A et al (2022) Consensus Definition of Misophonia: a Delphi Study. Front Neurosci :224 10.3389/fnins.2022.841816 PMC8969743 35368272

[16] Brout JJ (2022) A brief commentary on the Consensus Definition of Misophonia. Front Neurosci ;1019 10.3389/fnins.2022.879070 PMC9300890 35873831

[17] Naylor J, Caimino C, Scutt P, Hoare DJ, Baguley DM (2021) The prevalence and severity of misophonia in a UK undergraduate medical student population and validation of the Amsterdam misophonia scale. Psychiatr Q 92:609–619 32829440 10.1007/s11126-020-09825-3 PMC8110492

[18] Quek TC, Ho CS, Choo CC, Nguyen LH, Tran BX, Ho RC (2018) Misophonia in Singaporean psychiatric patients: a cross-sectional study. Int J Environ Res Public Health 15:1410 29973546 10.3390/ijerph15071410 PMC6069390

[19] Claiborn JM, Dozier TH, Hart SL, Lee J (2020) Self-identified Misophonia Phenomenology, Impact, and clinical correlates. Psychol Thought ;13

[20] Zhou X, Wu MS, Storch EA (2017) Misophonia symptoms among Chinese university students: incidence, associated impairment, and clinical correlates. J Obsessive Compuls Relat Disord 14:7–12

[21] Wu MS, Lewin AB, Murphy TK, Storch EA (2014) Misophonia: incidence, phenomenology, and clinical correlates in an undergraduate student sample. J Clin Psychol 70:994–1007 24752915 10.1002/jclp.22098

[22] Vitoratou S, Hayes C, Uglik-Marucha N, Pearson O, Graham T, Gregory J (2023) Misophonia in the UK: prevalence and norms from the S-Five in a UK representative sample. PLoS ONE 18. https://doi.org/10.1371%2Fjournal.pone.0282777 10.1371/journal.pone.0282777 PMC10032546 36947525

[23] Jakubovski E, Müller A, Kley H, De Zwaan M, Müller-Vahl K (2022) Prevalence and clinical correlates of misophonia symptoms in the general population of Germany. Front Psychiatry 13:2621 10.3389/fpsyt.2022.1012424 PMC9720274 36479555

[24] Vitoratou S, Uglik-Marucha N, Hayes C, Gregory J (2021) Listening to people with misophonia: explor- ing the multiple dimensions of sound intolerance using a new psychometric tool, the S-Five, in a large sample of individuals identifying with the condition. Psych 3:639–662. 10.3390/psych3040041

[25] Cronbach LJ (1951) Coefficient alpha and the Internal structure of tests. Psych 16:297–334. 10.1007/bf02310555

[26] Wicke FS, Krakau L, Löwe B, Beutel ME, Brähler E (2022) Update of the standardization of the Patient Health Questionnaire-4 (PHQ-4) in the general population. J Affect Disord 312:310–314 35760191 10.1016/j.jad.2022.06.054

[27] Hayes AF (2007) Li Cai. Using heteroskedasticity-consistent standard error estimators in OLS regression: an introduction and Software implementation. Behav Res Methods 39:709–722. 10.3758/bf03192961 18183883 10.3758/bf03192961

[28] Rouw R, Erfanian M (2018) A large-scale study of misophonia. J Clin Psychol 74:453–479 28561277 10.1002/jclp.22500

[29] Daniels EC, Rodriguez A, Zabelina DL (2020) Severity of misophonia symptoms is associated with worse cognitive control when exposed to misophonia trigger sounds. Langguth B, editor. PLOS ONE. ;15(1):e0227118 10.1371/journal.pone.0227118 PMC6964854 31945068

[30] Guetta RE, Cassiello-Robbins C, Trumbull J, Anand D, Rosenthal MZ (2022) Examining emotional functioning in misophonia: the role of affective instability and difficulties with emotion regulation. PLoS ONE 17:e0263230. 10.1371/journal.pone.0263230 35148347 10.1371/journal.pone.0263230 PMC8836307

[31] Smit DJ, Bakker M, Abdellaoui A, Hoetink AE, Vulink NC, Denys D (2022) Genetic evidence for the link of misophonia with psychiatric disorders and personality. medRxiv. 10.1101/2022.09.04.22279567 10.3389/fnins.2022.971752 PMC9902885 36760791

[32] Smit DJ, Bakker M, Abdellaoui A, Hoetink AE, Vulink N, Denys D (2023) A genome-wide association study of a rage-related misophonia symptom and the genetic link with audiological traits, psychiatric disorders, and personality. Front NeuroSci 16. 10.3389/fnins.2022.971752 10.3389/fnins.2022.971752 PMC9902885 36760791

